# Supplementary material for: Abnormal strong burn-in degradation of highly efficient polymer solar cells caused by spinodal donor-acceptor demixing
Source: Nat Commun. 2017 Feb 22;8:14541. doi: 10.1038/ncomms14541 (PMC5322537; doi:10.1038/ncomms14541)
Supplement: Supplementary Information — Supplementary Figures, Supplementary Tables and Supplementary References [file ncomms14541-s1.pdf]

Supplementary Table 1 photovoltaic parameters of fresh and aged PCE11:PCBM solar cells.

	V_{OC} [V]	J_{SC} [mA cm^{-2}]	FF [%]	PCE [%]
**Fresh**	0.74	17.84	69.7	9.20
**Aged**	0.73	11.78	65.4	5.62

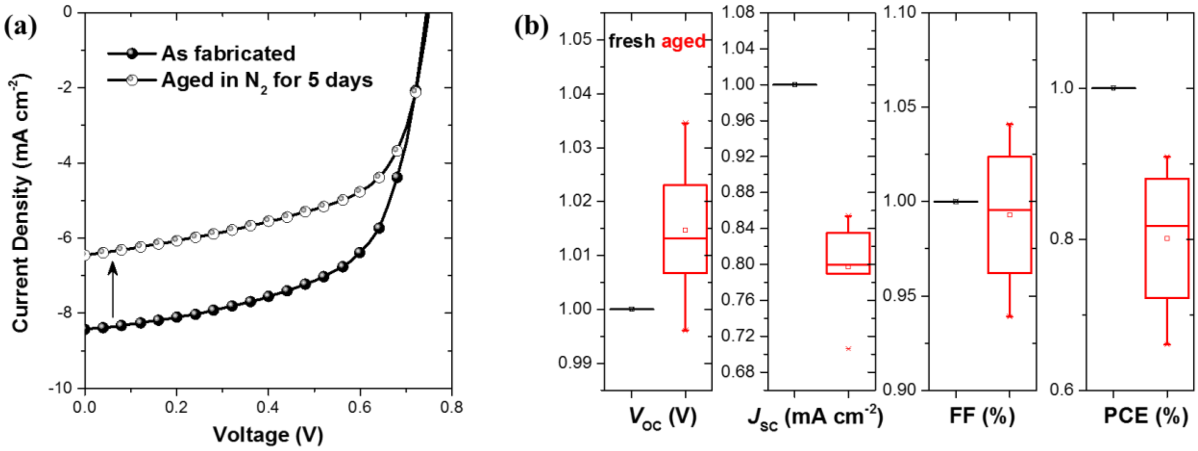

Supplementary Figure 1 (a) *J-V* characteristics of PCE11:PCBM OSCs measured after fabrication (fresh) and aged in N_2 for 5 days (aged). The PCE11:PCBM OSCs were fabricated without DIO. (b) Normalized photovoltaic parameters of 12 PCE11:PCBM (without DIO) aged samples. The values measured after fabrication (fresh) are normalized to 1.

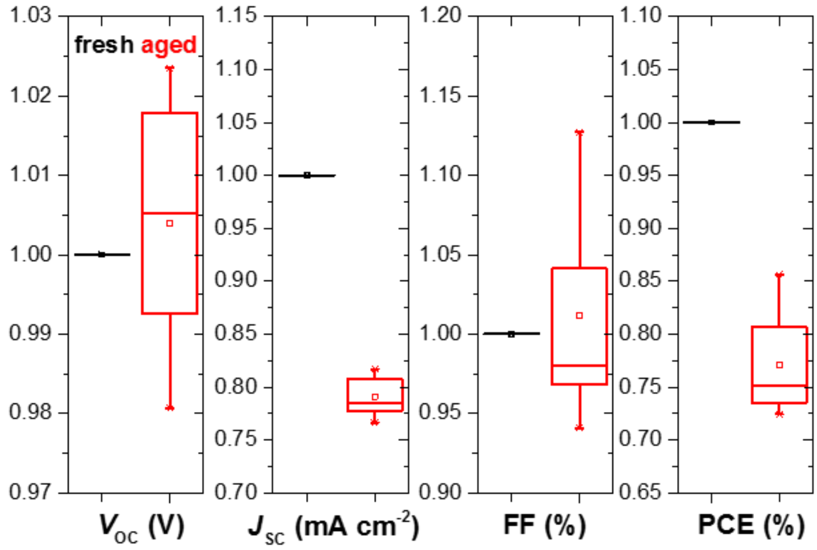

Supplementary Figure 2 Normalized photovoltaic parameters of 12 PCE11:PCBM (encapsulated in N_2) aged samples. The values measured after fabrication (fresh) are normalized to 1.

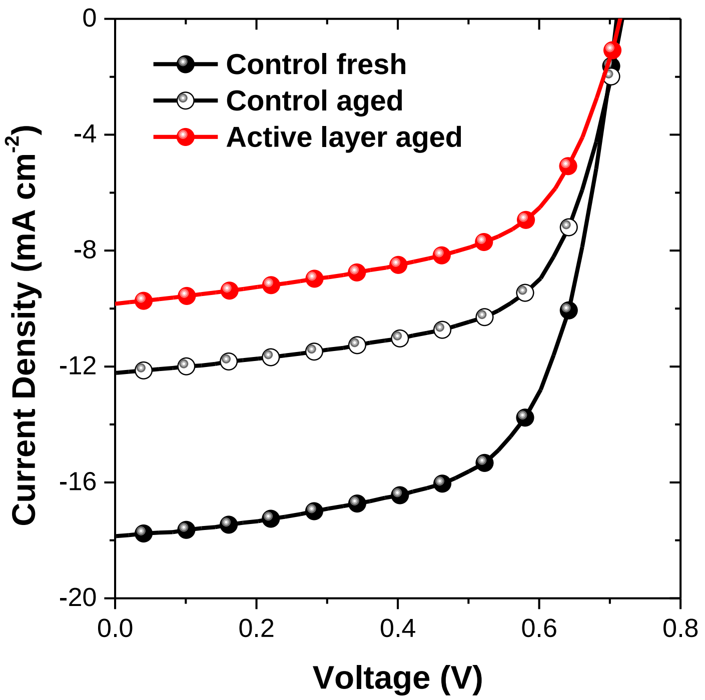

Supplementary Figure 3 *J-V* characteristics of optimized PCE11:PCBM solar cells that were aged in air for 5 days with (control device) and without (active layer aged) the top MoOx/Ag electrode.

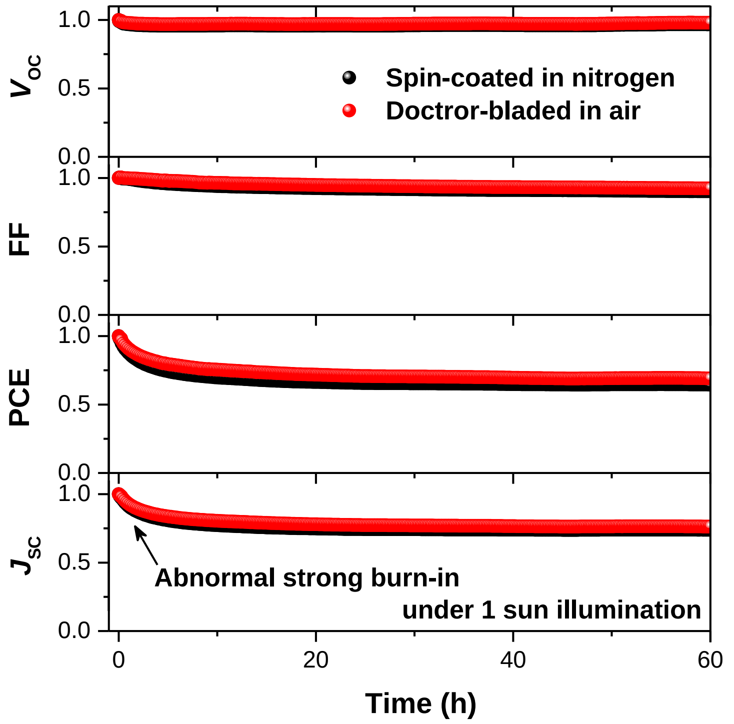

Supplementary Figure 4 Evolution of photovoltaic parameters of an optimized PCE11:PCBM solar cell measured under continuous 1 sun illumination. The devices fabricated by spin-coating in nitrogen were treated without exposure to air.

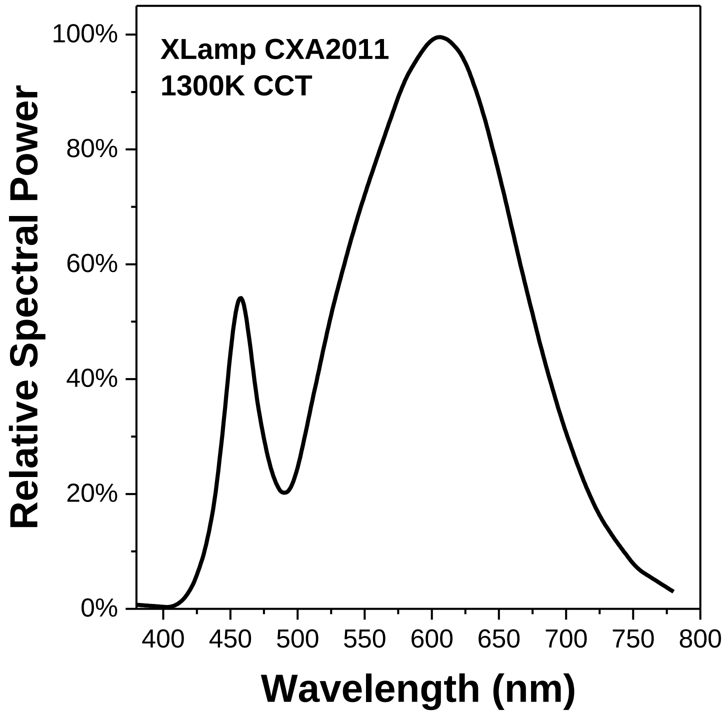

Supplementary Figure 5 Illumination spectrum of the white LED used for the stability test.

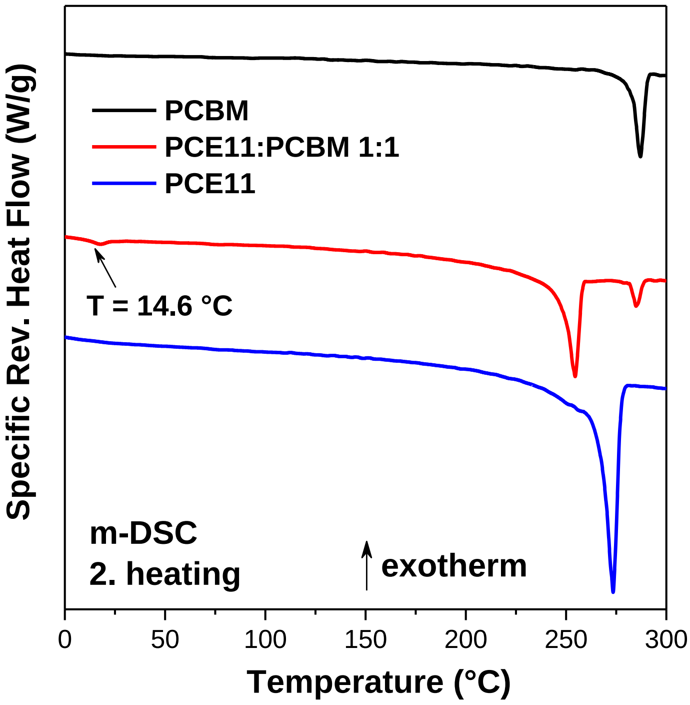

Supplementary Figure 6 The 2nd heating scans of PCE11, PCBM and PCE11:PCBM 1:1 blend measured by temperature modulated DSC.

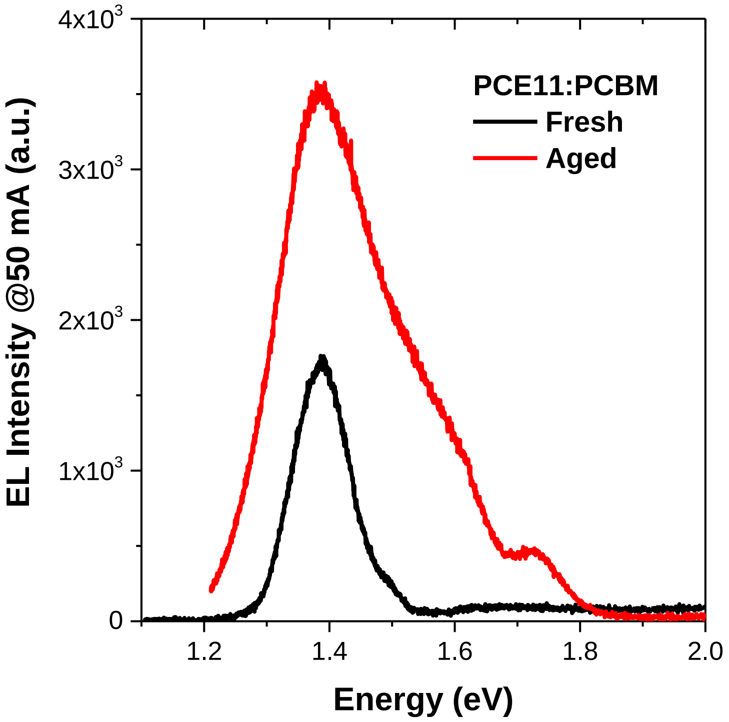

Supplementary Figure 7 EL spectra of fresh and aged PCE11:PCBM samples measured at an external constant current of 50 mA.

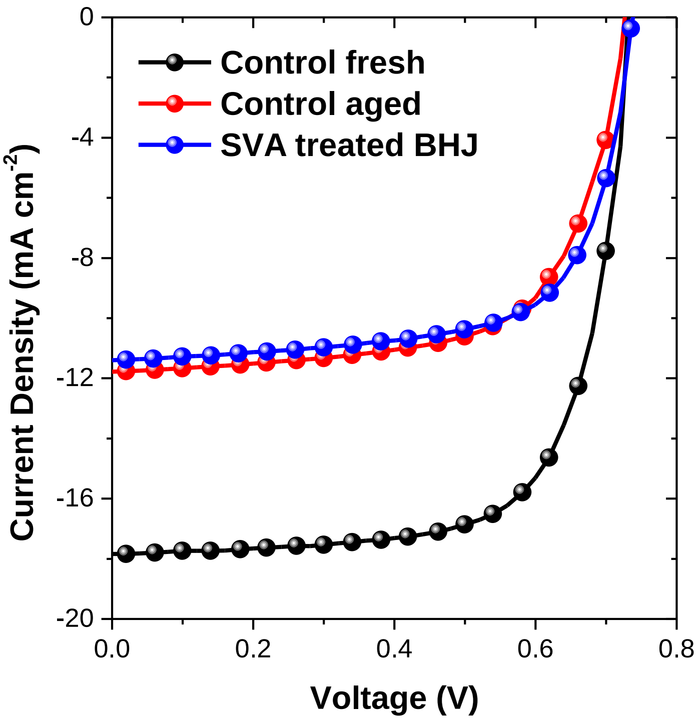

Supplementary Figure 8 *J-V* characteristics of optimized PCE11:PCBM control devices and devices based on solvent vapor annealing (SVA) treated BHJ active layer (average over 20 devices).

Supplementary Table 2 Fitting parameters used for modeling the CT absorbance band of fresh and aged PCE11:PCBM. The CT region of PCE11:PCBM was fitted with the GaussAMP function using the equation[1] $y = Ae^{-\frac{(x-x_C)}{2w^2}}$ where A is the amplitude, x_c is the centroid and w is the width of the fitting. The value of x_c was taken from the peak maximum of EL spectra, which is located at 1.39 eV.

	Fresh PCE11:PCBM	Aged PCE11:PCBM
x_c	1.39	1.39
**w**	0.1	0.1
**A**	0.006	0.0015

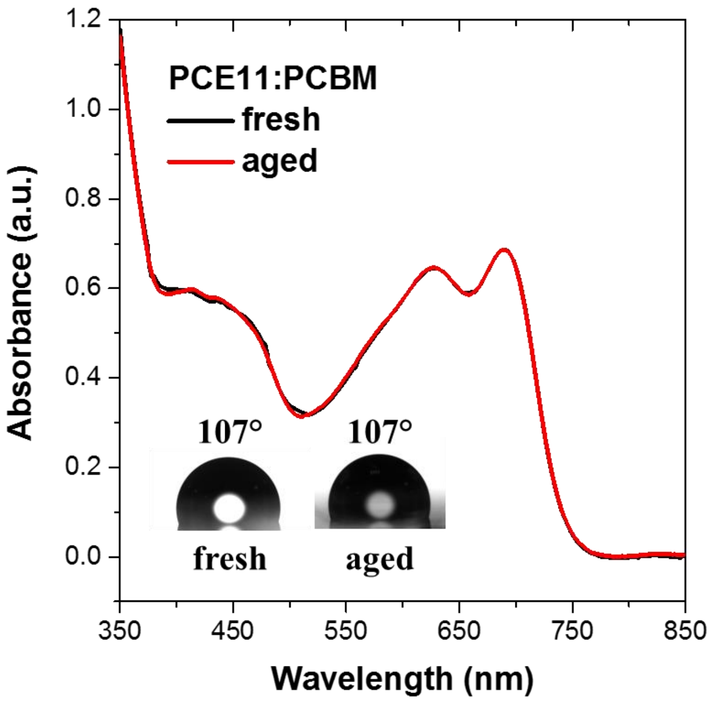

Supplementary Figure 9 Absorption spectra of PCE11:PCBM fresh and aged samples. Inset pictures illustrate the contact angle of water droplet on the corresponding active layers.

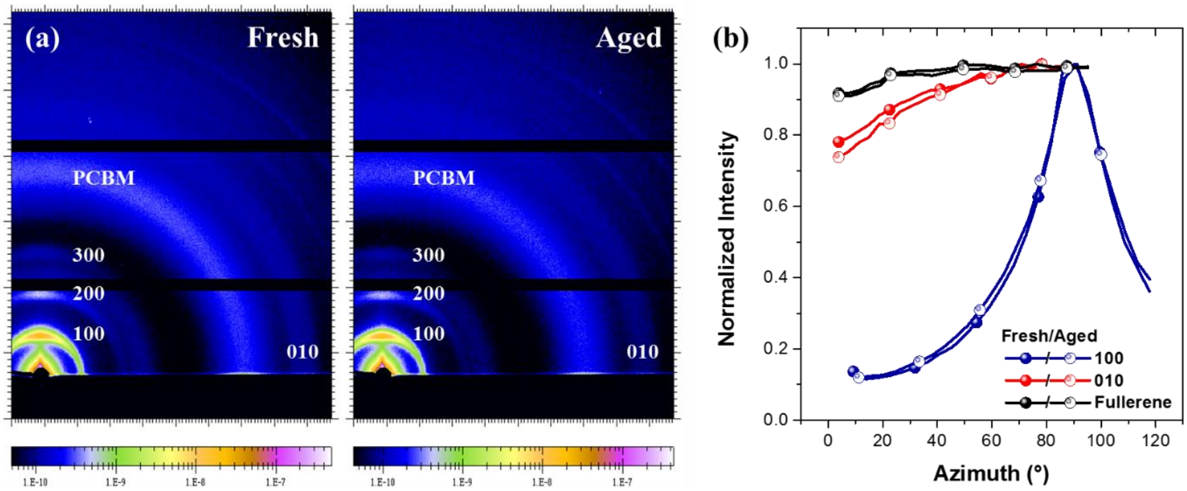

Supplementary Figure 10 The 2D GIWAXS patterns (a) and the corresponding azimuth information (b) of PCE11:PCBM fresh and aged samples. The colour bars represent the intensity of the GIWAXS data.

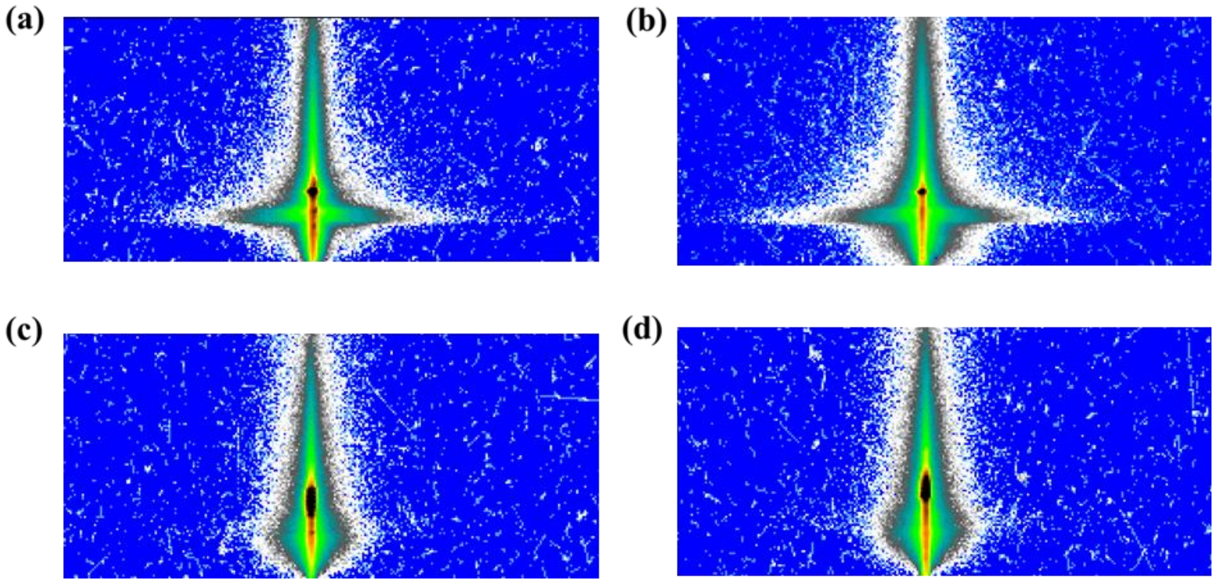

Supplementary Figure 11 The 2D GISAXS patterns of (a) fresh PCE11:PCBM, (b) aged PCE11:PCBM, (c) fresh neat PCE11, and (d) aged neat PCE11 samples.

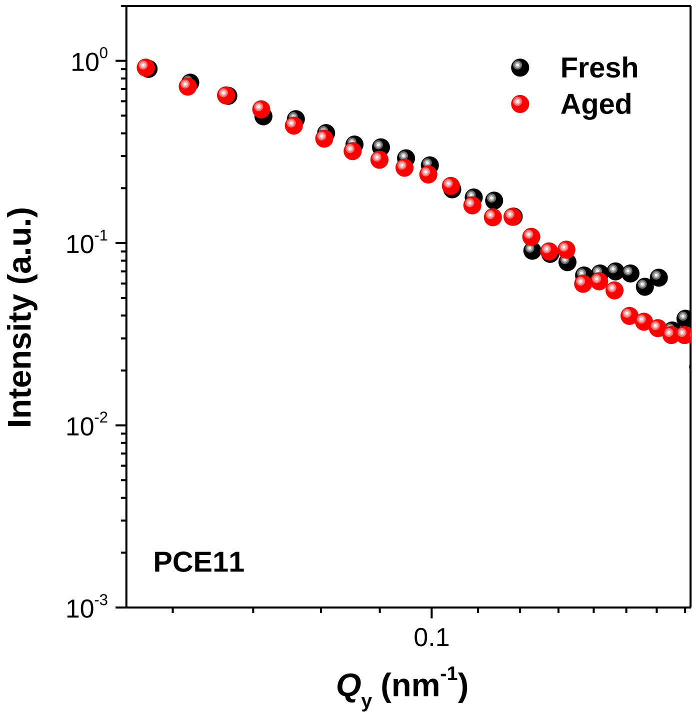

Supplementary Figure 12 The GISAXS profiles of fresh and aged neat PCE11 collected from in-plane cuts made at an exit angle equal to the Yoneda peak of the active layer.

Supplementary Table 3 Structural parameters determined by model-fitting of GISAXS profiles of PCE11:PCBM thin films.

	Correlation length of the mixed region (nm)	Fullerene clusters		
		Average domain size (nm)	Width of size distribution (nm)	Volume fraction %
Fresh	7.71109	43	0.208399	6.87
Aged	8.16728	78	0.872295	36.8322

Supplementary Table 4 Enthalpy change of PCE11, PCBM and PCE11:PCBM 1:1 blend. [(1)] Melting peak at low temperature. [(2)] Melting peak at high temperature.

	**PCE11**	**PCBM**		**PCE11:PCBM**	
		**(1)**	**(2)**	**(1)**	**(2)**
**1. Heating**	30.24 J/g	7.298 J/g	18.05 J/g	11.85 J/g	7.016 J/g
**2. Heating**	24.08 J/g	2.427 J/g	10.14 J/g	10.90 J/g	2.984 J/g
**1. Cooling**	25.56 J/g	9.605 J/g		17.13 J/g	

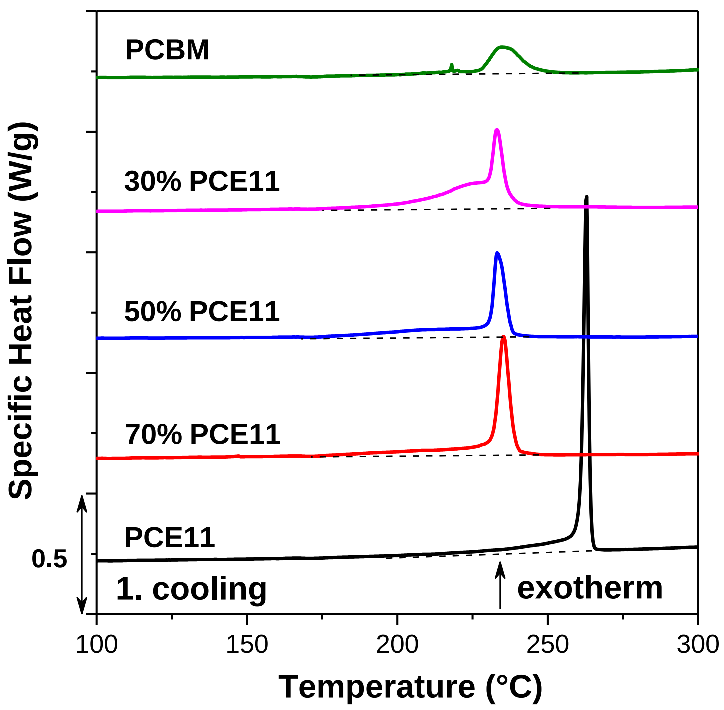

Supplementary Figure 13 The 1st cooling scans of PCE11:PCBM blends.

Supplementary Table 5 Enthalpy change ΔH of PCE11:PCBM blends and neat materials extracted from the 1st cooling scan.

	**ΔH**
**PCE11**	25.56 J/g
**70% PCE11**	19.74 J/g
**50% PCE11**	17.13 J/g
**30% PCE11**	21.95 J/g
**PCBM**	9.605 J/g

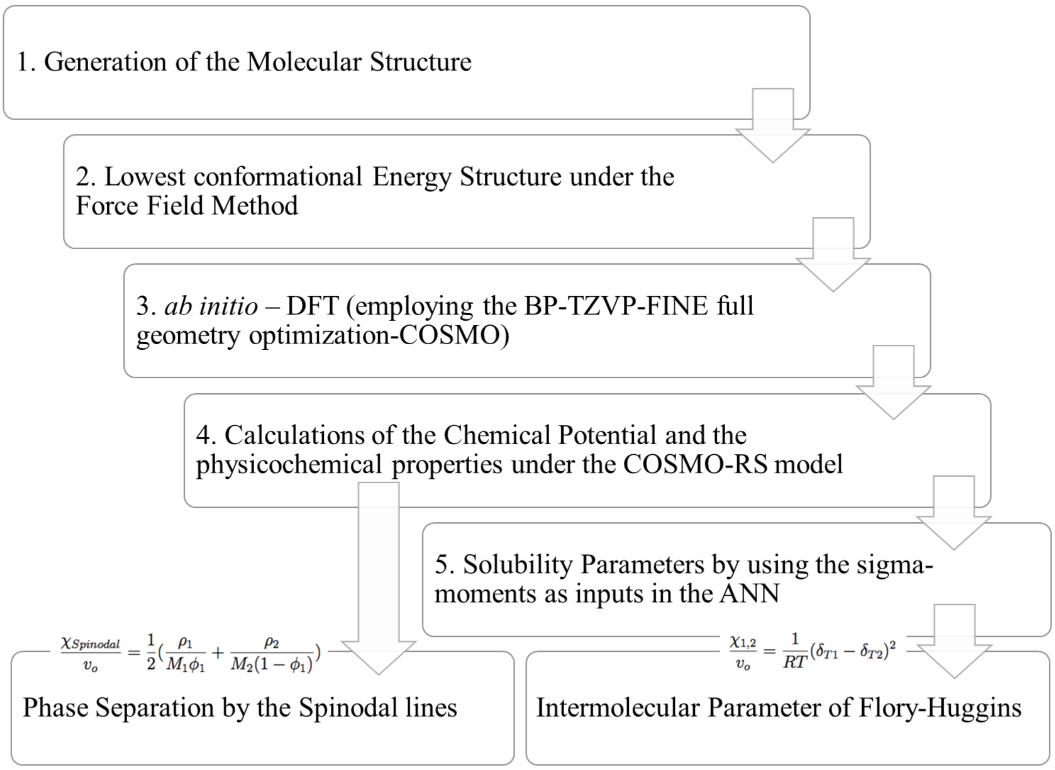

Supplementary Figure 14 Workflow of the theoretical calculation used for predicting the phase behavior and miscibility of donor and acceptor systems.

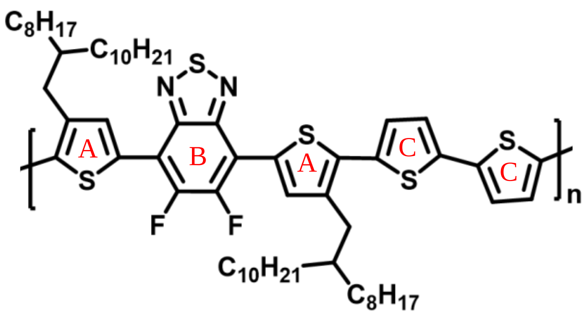

Supplementary Figure 15 Moieties molecular structure of PCE11 used for estimating its molecular weight and molecular volume. Different from the homo-polymer P3HT, the repeating unit of copolymer PCE11 consists of 5,6-difluorobenzothiadiazole and quarterthiophene moieties. To estimate the molecular weight (MW) and molecular volume (MV) of PCE11 in a reasonable way, the molecular weight and molecular volume were calculated as follows: $\mathrm{MW}_{PCE11} = 0.4 * \mathrm{MW}_A + 0.2 * \mathrm{MW}_B + 0.4 * \mathrm{MW}_C$ and $\mathrm{MV}_{PCE11} = 0.4 * \mathrm{MV}_A + 0.2 * \mathrm{MV}_B + 0.4 * \mathrm{MV}_C$.

Supplementary Table 6 The calculated molecular weight, molar volume, liquid density and Hildebrand parameter δ_T for P3HT, PCE11, PCBM and ICBA. * Experimental values estimated in our lab or in literature.[2-4]

Molecule	Molecular Weight (g mol^{-1})	Molecular Volume (cm^3 mol^{-1})	Liquid Density (g cm^{-3})	δ_T (MPa$^{1/2}$)
P3HT	173.80/166.1*	148.668/151.0*	1.17/1.1*	19.36
PCE11	226.354	187.168	1.21	19.14*
PCBM	910.89/910.5*	548.041/607.0*	1.66/1.5*	21.60
ICBA	936.84/952.5*	542.652/635.0*	1.72/1.5*	20.81

Supplementary Table 7 Interaction parameter $\chi_{1,2}$ determined for P3HT:PCBM, P3HT:ICBA, PCE11:PCBM and PCE11:ICBA.

Molecules	Calculated $\chi_{1,2}/v_0$
P3HT:PCBM	0.00202
P3HT:ICBA	0.00085
PCE11:PCBM	0.00244
PCE11:ICBA	0.00112

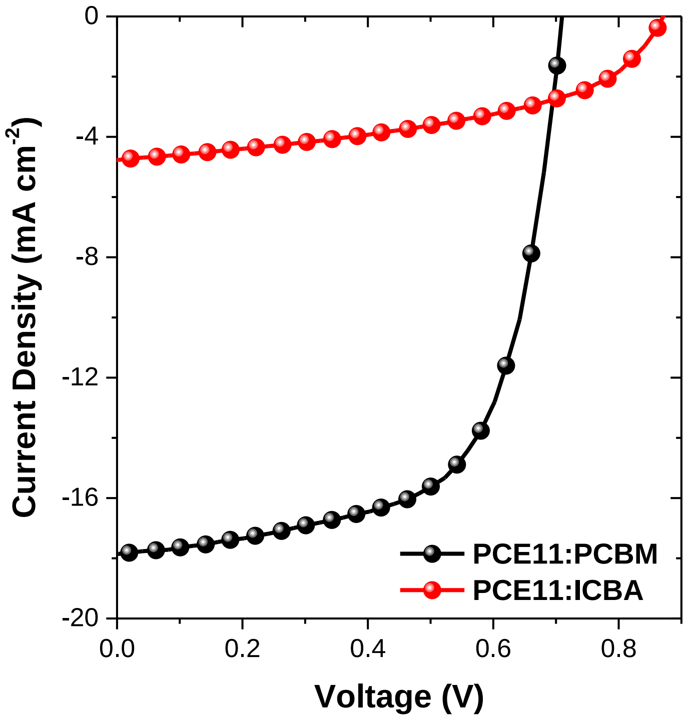

Supplementary Figure 16 *J-V* characteristics of PCE11:PCBM and PCE11:ICBA solar cells.

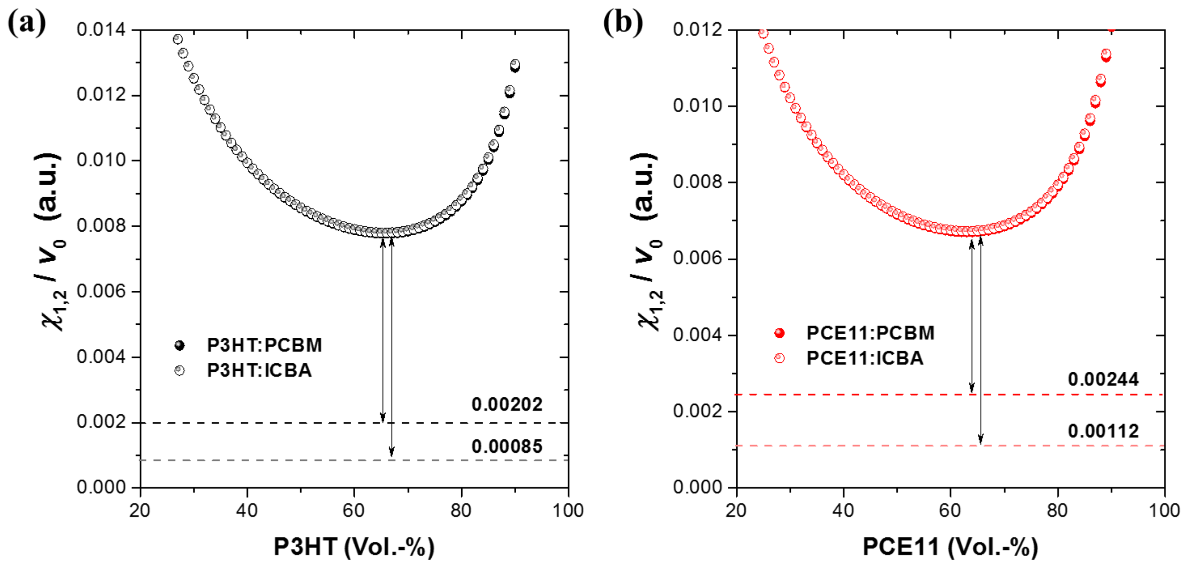

Supplementary Figure 17 The polymer/fullerene liquid (melt) solid transition diagrams estimated for P3HT:PCBM, P3HT:ICBA, PCE11:PCBM and PCE11:ICBA as a function of the volume fraction of polymer. The dashed lines represent the interaction parameters of polymer-fullerene blends.

Supplementary References

1 Vandewal, K. *et al.* The Relation Between Open-Circuit Voltage and the Onset of Photocurrent Generation by Charge-Transfer Absorption in Polymer : Fullerene Bulk Heterojunction Solar Cells. *Advanced Functional Materials* **18**, 2064-2070, (2008).

2 Perea, J. D. *et al.* Combined Computational Approach Based on Density Functional Theory and Artificial Neural Networks for Predicting The Solubility Parameters of Fullerenes. *The Journal of Physical Chemistry B* **120**, 4431-4438, (2016).

3 Ulum, S. *et al.* The role of miscibility in polymer: fullerene nanoparticulate organic photovoltaic devices. *Nano Energy* **2**, 897-905, (2013).

4 Kozub, D. R. *et al.* Polymer crystallization of partially miscible polythiophene/fullerene mixtures controls morphology. *Macromolecules* **44**, 5722-5726, (2011).
